# Climate Change and Cerebrospinal Meningitis in the Ghanaian Meningitis Belt

**DOI:** 10.3390/ijerph110706923

**Published:** 2014-07-07

**Authors:** Samuel Nii Ardey Codjoe, Vivian Adams Nabie

**Affiliations:** Regional Institute for Population Studies, University of Ghana, P.O. Box LG 96, Legon, Ghana; E-Mail: nabievivian@gmail.com

**Keywords:** climate change, cerebrospinal meningitis, adaptation, meningitis belt, Ghana

## Abstract

Cerebrospinal meningitis (CSM) is one of the infectious diseases likely to be affected by climate change. Although there are a few studies on the climate change-CSM nexus, none has considered perceptions of community members. However, understanding public perception in relation to a phenomenon is very significant for the design of effective communication and mitigation strategies as well as coping and adaptation strategies. This paper uses focus group discussions (FGDs) to fill this knowledge lacuna. Results show that although a few elderly participants ascribed fatal causes (disobedience to gods, ancestors, and evil spirits) to CSM infections during FGDs, majority of participants rightly linked CSM infections to dry, very hot and dusty conditions experienced during the dry season. Finally, community members use a suite of adaptation options to curb future CSM epidemics.

## 1. Introduction

It is now abundantly clear that climate change is occurring globally [1], and having impacts on ecological systems worldwide [2]. It has therefore been suggested that attention should be given to studies that considers the link between climate change and infectious diseases, so as to have a better understanding of the nature of the relationship [3,4]. Cerebrospinal meningitis (CSM) also referred to as meningococcal meningitis is one of such infectious disease likely to be affected by climate change [1,5]. 

There are a few studies on the climate change-CSM nexus, these studies have generally been on models predicting epidemics and thus mapping risk areas [6,7,8,9,10,11], or reviews of past studies [12,13]. While other studies have been on the extension of the meningitis belt [14], others have been undertaken in the northern hemisphere where similar seasonal incidence compared to the African meningitis belt exists [15,16,17,18,19,20,21,22]. Furthermore, quite a substantial proportion of the studies have considered the seasonality of epidemics. Thus, it has been stated that epidemics stop with the onset of the rainy season, and resume in the dry season [23,24,25,26]. In addition, the disease is linked with humidity [27,28,29], rainfall [30], dry harmattan winds and dusty conditions [31,32,33,34].

Recommendations for future research on CSM include immunity in populations, carriage rates, vaccination coverage, social interactions and emergence of new strains of the disease [7,10]. Furthermore, since the meningitis belt has expanded and all the signs are there that it will further expand as a result of climate change, studies have recommended that close surveillance of CSM incidence in the areas bordering the meningitis belt should be carried out to allow for early detection [9]. In addition, molecular biology studies to understand the effects of climate change on the binding and penetration of *N. meningitis* in mucosal membranes have also been recommended. Finally, development of early warning systems to include demographic data, data on vaccination coverage and natural immunity in populations and bacteriological surveillance data on the strains to predict epidemics in time for effective mass vaccination campaigns have also been recommended [11]. 

It is obvious in the review of studies and recommendations above that there is no study to comprehend the nature of the climate change-CSM nexus from the point of view of community members through public perception studies. However, it has been predicted that community members in settings such as the one being considered in this paper are the ones that will experience the severest impacts from climate change. In addition, understanding public perception in relation to a phenomenon is very significant for the design of effective communication and mitigation strategies as well as coping and adaptation strategies [35,36,37,38]. This paper attempts to fill this knowledge lacuna by understanding the nature of the climate change-CSM nexus from perceptions of community members.

### 1.1. Overview of Cerebrospinal Meningitis

**Figure 1:**
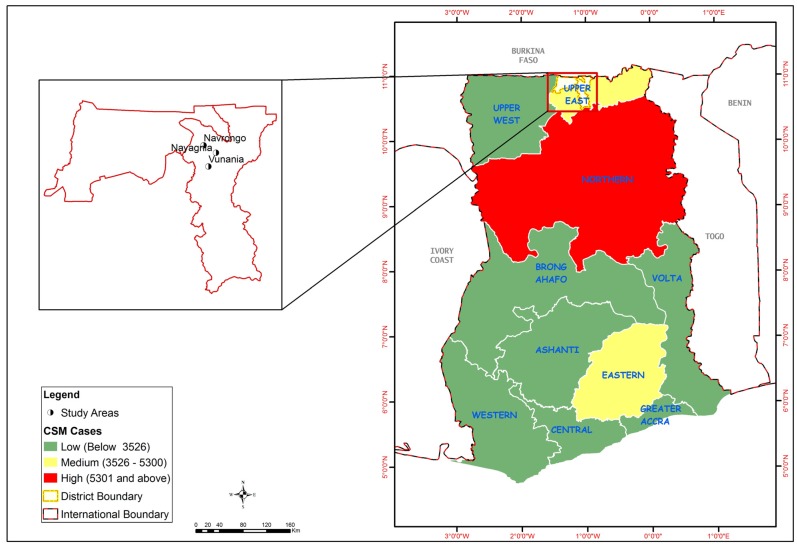
Map of Ghana showing regional distribution of recorded CSM cases, 1985–2008, and the study area.

Note: Source: Ministry of Health [42].

CSM is a respiratory and contagious disease. Clinical CSM disease was first described by Vieusseux in 1805 in Geneva, Switzerland while the causative agent, *Neisseria meningitidis* was identified by Austrian bacteriologist Anton Weichselbaum in 1887 [39]. Since then, the disease has occurred in epidemic proportions in other parts of the world including China, Vietnam, Mongolia, Saudi Arabia and Yemen, Europe and the Americas [40]. The first report of a meningitis epidemic in Africa occurred in 1840. African epidemics thereafter became much more common in the 20th century in the African meningitis belt [41]. In Ghana, CSM cases have been recorded in all the regions, with Northern region recording the highest cases. The study area is located in the Upper East region which is a medium endemic area (Figure 1). 

Bacterial meningitis is an infection or inflammation of the protective membranes covering the brain. Several different bacteria are however said to possess the potential to cause bacterial meningitis and *Neisseria meningitidis* is one of the most important because of its potential to cause epidemics [40] (the other bacteria, besides *N. meningitidis* (meningococcus) that can cause bacterial meningitis include *S. pneumoniae* and *H. influenzae* type b). Bacterial meningitis is now said to be among the top 10 infectious causes of death worldwide [43]. Twelve subtypes or serogroups of *N. meningitidis* have been identified and four (*N. meningitidis A, B, C* and *W135*) are recognized to cause epidemics [33,44]. Meningeal syndrome, the septic form and pneumonia are the three main clinical forms of the disease. 

The bacteria causing CSM has been identified to be transmitted from person to person through droplets of respiratory or throat secretions [40]. The bacteria can be carried in the pharynx and it overwhelm the body’s defences. This allows infection to spread through the bloodstream and to the brain. Conditions that favour transmission of the bacteria are densely populated rooms with inadequate ventilation, overcrowding at public events including worship centres and markets, as well as population movements during pilgrimages [9,41,45]. Incubation period ranges from 2 to 10 days with an average of 4 days, and *N. meningitidis* does not infect animals but only humans [39].

Finally, climatic conditions characterised by dry winds, dust storms, low humidity and cold nights considerably diminishes the local immunity of the pharynx thereby increasing the risk of meningitis [28,29,33,34,46,47,48,49].

CSM has varying symptoms and the most common ones are stiff neck, high fever, sensitivity to light, convulsion, headaches and vomiting [39]. Even when the disease is diagnosed early and adequate therapy instituted, 5% to 10% of patients still die, typically within 24–48 h of onset of symptoms [40]. In the event that a person survives, bacterial meningitis may result in brain damage, hearing loss or learning disability in 10% to 20% of survivors. 

In 1996, Africa experienced the major recorded outbreak of epidemic meningitis in history, with over 250,000 cases and 25,000 deaths registered. Between that crisis and 2002, 223,000 new cases were reported to the World Health Organization [39,50,51,52]. The countries most affected during this period had been Burkina Faso, Chad, Ethiopia and Niger. In 2002, the outbreaks occurring in Burkina Faso, Ethiopia and Niger were said to have accounted for about 65% of the total cases reported on the African continent. Furthermore, the meningitis belt has been observed to be extending further south. In 2002, the Great Lakes region of east Africa was affected by outbreaks in villages and refugees camps which caused more than 2200 cases, including 200 deaths [52]. 

### 1.2. Cerebrospinal Meningitis in Ghana

CSM studies in Ghana dates back to the 1900s when it was first discovered [53]. Initial studies mainly concentrated on mortality cases as a result of the disease. The first recorded outbreak of CSM was in Cape Coast in southern Ghana in 1900 among East African labourers who were brought to the Gold Coast to support the British campaign against the Ashanti of central Ghana [54]. This outbreak died out rapidly without causing an epidemic in the local population. The next epidemic in the Gold Coast started in 1906 from the north-west and spread through the northern territory during the following dry season. Estimates show that the disease was responsible for the deaths of 20,000 people between 1906 and 1908 [55]. Since that period, epidemics were recorded every 8–12 years [54,56,57,58]. Thus, records have shown that the morbidity rate of the disease for a single year has ranged from a low of 8 cases near Bole in northern Ghana in 1944 [59], to a high of 18,703 cases in the entire country in 1997 [60,61,62]. These deaths have occurred at the peak of the harmattan in the three northern regions of Ghana (Upper East, Upper West and Northern) with a marked decline by the end of April. 

A whole array of studies considers risk factors, survival and sequelae issues associated with meningitis in Ghana [63,64,65]. Other studies have investigated different serogroups associated with the disease [66,67,68,69,70], and the emergence of a particular serogroup. *i.e.*, W-135 linked with returned Hajj pilgrims [71]. Emergency vaccination against epidemics of the disease [62] has also received attention in the literature. Furthermore, some studies have looked at the bacteria that cause meningitis in children [72,73]. More recent studies have considered knowledge, attitude and practice related to the disease [74], economic burden of the disease to households in Ghana [75], and assessed association between number of reported meningitis cases and a set of weather variables [76]. This study has two main objectives: (i) assess the public perception of the climate change-CSM nexus; and (ii) investigate the local adaptation strategies used. 

### 1.3. Study Area

The Kassana-Nankana East Municipality (KNEM) of the Upper East Region of Ghana is used for this study (Figure 1). According to the Ghana Statistical Service [77], the population of KNEM in 2010 was 109,944, and it is generally rural except for those living in Navrongo, the municipal capital. Households live in dispersed compound houses with an average size of 5.4. KNEM has a sub-Sahelian climate of a long dry season and a short rainy season. The dry and wet seasons, are mainly influenced by two air masses—the North-East Trade Winds (Harmattan Air Mass) and the South-Westerlies (Tropical Maritime Air Mass). The north-east Trade Winds is usually dry and dusty as it originates from the Sahara Desert. During this season (November to April), rainfall is virtually absent due to low relative humidity, which rarely exceeds 20%, and low vapour pressure of less than 10 mb. Day temperatures are high, rising up to 42 °C in February and March, and night temperatures fall as low as 18 °C. KNEM experiences the South-Westerlies between May and October with rainfall averaging 950 mm per annum. KNEM has one hospital located in Navrongo which serves as the referral point to all health centres. There are two health centres; one health post; one private clinic and fourteen pharmacy shops. 

## 2. Methodology

### 2.1. Data

The study used data generated from focus group discussions (FGDs) undertaken in Vunania and Nayagnia, both located to the north-west of the municipality, and very close to Navrongo, the municipal capital The two communities were selected because they recorded high incidence of CSM in the past, which is now declining. Six focus group discussions were conducted; three each in each of the two study communities. A total of 60 participants selected by other community members to represent them during earlier community meetings took part in the FGDs. The participants were grouped into adult males, adult females and youth. Both male and female adults are aged 35 years and above while the ages of the youth ranged from 15 to 34 years. A total of 8–12 participants were in each group. Out of the total of 60 FGD participants, 35 are males and 25 females. Regarding age, nine were aged below 25 years, 14 aged between 25 and 30 years, 19 aged between 35 and 40 years and 18 were 45 years or older. Furthermore, 29 of the participants have no formal education, 21 have basic education, nine have secondary education, and only one participant has tertiary education. Finally, 24 of the participants were farmers, 20 are traders, six are day labourers, one is a professional and nine are unemployed. In addition, 10 key informant interviews were conducted with the medical superintendent of the War Memorial Hospital; health officials, disease control officers and assembly members.

Using a pre-designed focus group discussion guide, the discussions were conducted under the guidance of a facilitator and a note-taker with the help of a tape recorder. The discussions were on perceptions of participants on the link between climate change and CSM, and adaption strategies used. The FGDs were conducted in *Kassin*, the local language. Prior to all these activities, preliminary visits were undertaken to obtain the informed consent from FGD participants and community leaders. 

### 2.2. Method of Analysis

Qualitative data from FGDs was analysed using the thematic framework. This process involved a series of steps: familiarization, and identification of thematic frameworks; indexing; charting; mapping and interpretation. In getting familiarized with the information gathered, audio tape was carefully listened to and the transcripts and observational notes taken during the discussions were read in their entirety. This was to identify the dominant themes that emerged during the discussions. In indexing, a detailed analysis of the transcript was undertaken making use of codes from the responses obtained in order to sort out interesting quotes from the transcripts with the aid of NviVO coding. 

Through this, a determination was made of the emerging themes in line with the objectives of the study. Subsequently, a coding frame was developed and the coded data was mapped and interpreted. In doing this, particular attention was paid to the context, comments, and words in order to enhance the internal consistency of the data. Triangulation techniques, in the form of discussions with key community leaders were used to reduce potential bias effects in the data.

## 3. Results and Discussion

### 3.1. Public Perceptions of Climate Change and CSM Prevalence

Analysis show that participants generally displayed a high level of awareness of variations in climatic variables mainly temperature and rainfall in their communities. It was stated that the duration of the hot dry season is now much longer than it used to be. It now spans from February to June instead of from March to May in the past. Participants stated that the heat experienced in recent times is much more severe than in the past since temperatures could now rise to 48 °C. A female participant in Nayagnia stated the following:

*The weather is now not stable and I cannot predict when the seasons will start and end. Some years ago, the rains would start in early May as the latest and end in October, then the harmattan season will start in October and end around February, and the dry season continues from March to late May. But now I cannot tell. This is seriously affecting us. Because we rely on the rains for farming and the dry season to sell our farm produce. Besides, the temperature during the dry season is increasing every year.*

Regarding rainfall, participants stated that the number of rainy days and volume of rainfall being experienced now have both seen significant changes compared to the past. In their view, the number of rainy days has generally reduced, although occasionally the rains last until the end of October. They recalled the year 2012 when the rains started in June and lasted until end of October. The volume of rainfall in that year was extraordinarily high. The sentiment above was summarized by a youth participant in Nayagnia as follows:

*The rain does not fall at the correct time these days. It will fall at a time when you don’t expect it to fall. These days it also falls very much when we don’t expect it. I think, t* *he rainfall pattern has changed. At one time you experience very heavy rainfall over a very long time which leads to floods, and at other times it only drizzles over a couple of days, causing droughts and dusty conditions. These are the challenges we are facing in this community and we cannot do anything about it.*

Participants also associated the phenomenon of climate change to the growing incidence of droughts and floods that have characterised recent times. In their view, floods are becoming an annual phenomenon. This is corroborated by studies by Tschakert, Sagoe, Ofori-Darko, and Codjoe [78]. The recent floods are linked to the opening of the Bagre Dam in neighbouring Burkina Faso, which suffers from excessive rains, which invariably results in spillage. The officer at the Navrongo Meteorological Station stated the following:

*Climate change is real in Navrongo. Over the course of the 25 years that I have worked at this station, I have observed a progressive rise in temperature and decrease in mean annual rainfall. Climate change is therefore happening all around us due to rising temperatures; declining rainfall totals and increased variability; high incidence of weather extremes and disasters.*

Participants’ knowledge and level of awareness of the incidence of CSM are also ascertained. Participants generally held the view that CSM is a disease that mostly affects people during the dry season; adding that the disease only occurs when the weather is hot. Furthermore, other participants including the district disease control officer stated that the disease occurs in epidemic proportions when there is an incidence of drought, which comes along with severe heat and dusty conditions.

*Most communities here are prone to CSM because of their location near the Sahelian region, which is CSM-endemic. CSM mostly assumes endemic proportions when the Ghana Health Service fails to vaccinate people before the start of the dry season. In that case, the heat and congestion in most compounds serve as a conducive atmosphere for the disease to thrive given that the people possess very little capacity to prevent the occurrence of the disease.*

Notwithstanding the significant levels of knowledge and awareness displayed by the participants on the causes of CSM, a few of the older participants ascribed fatal causes to the incidence of CSM. In the view of an elderly male participant in Vunania, CSM is a disease that is caused and spread by their ancestors as punishment for disobedience.

*Well, everybody here can associate CSM with temperature and rainfall, but some of us still believe that the disease is a curse from our forefathers on people who fail to honour them or those who disobey the gods. I remember a young boy once insulted the gods when he was brought before them to apologise for an offense he had committed. He refused to apologise and before we knew it he was afflicted by the disease and he died. Some of us still believe that the disease is from our ancestors. Once we stop offending them, they will also stop infecting our children and generations yet to come with the disease.*

Participants commented on the link between climatic change and CSM incidence. They were unanimous in their opinion that the recent changes in temperature which subsequently results in severe heat, dusty and drought conditions, as well as recent decreases in rainfall, combined with considerably high room occupancy rates, poor ventilation and dehydration is responsible for increases in CSM epidemics. The following opinion was expressed by a male participant in Nayagnia.

*For us we have observed that in recent years the heat becomes so severe, and that is when we experience a lot of CSM cases. It happened some few years back when the meteorological officer told us that temperatures for the first time went up to 48 °C. A number of people in this community were attacked by the disease.*

A female participant in Vunania expressed the following opinion.

*Unlike malaria which occurs at any time of the year, CSM is the only disease which attacks us during the dry season, and in years when the heat becomes very severe. One thing we still do not understand is that the disease also affects us the poor people and our children.*

### 3.2. Adaptation Strategies

Adaptation has been defined as the “adjustments in ecological, social, and economic systems in response to actual or expected climatic stimuli and their effects or impacts, which moderates harm or exploits beneficial opportunities” [79]. Adaptation strategies could be autonomous or planned, and reactive or anticipatory [80]. Autonomous adaptation pertains to spontaneity in the response while conscious interventions are made in planned adaptation [81]. While deliberate decisions to prepare for potential adverse impacts before a climatic event or trend occurs are made in anticipatory adaptation, reactive adaptation is undertaken after the event or trend has been observed. Adaptation could also be either short-term or long-term. It is important to note that adaptation strategies are not mutually-exclusive to these categories, and one adaptation strategy could belong to more than one category.

#### 3.2.1. Reactive Adaptation: Annual Mass CSM Immunization, Moderation of Socio-cultural Activities that Result in Mass Gathering and Movement Restrictions on Carriers

Three adaptation strategies that are reactive were mentioned by FGD participants. Firstly, mass immunization programmes undertaken by the Ghana Health Service targeted at endemic areas during outbreaks was mentioned. Prior to 2012, Polysacharide A, C and W-135 vaccines were used, but currently, PsA-TT also known as MenAfriVac is provided. Participants noted that in years when immunizations are done early enough, the incidence rate of CSM is drastically reduced as stated below by a male participant in Vunania.

*We thank the government for the annual vaccination which is done in the dry season against CSM. This is helping us and saving the lives of our children, brothers, sisters and community members who used to die when there is an outbreak. Now we are not afraid of the disease like in the past.*

This finding has been confirmed by Forgor [53] revealing that early vaccination has the potential to reduce by up to 80 percent the incidence rate of the disease. The challenge with immunization, as observed by a male participant in Vunania is that when it is undertaken in the course of a CSM epidemic outbreak, it only slightly reduces the impacts.

*The vaccinations have been very helpful in fighting the disease in this community, especially when they do it before the beginning of the dry season. They are however not able to solve the problem completely when they delay in the vaccination before the outbreak occurs. I say this because my son died of the disease whilst on admission at the hospital in Navrongo some ten years ago.*

Though mass CSM immunization is identified and used as the main strategy to reduce the incidence of CSM in recent times, participants also stated that a segment of the population is against these vaccinations, and hence do not make themselves and families available for immunization. They are of the opinion that as already mentioned the disease is caused by the gods and ancestors. Thus, since they do not offend the gods and ancestors, they will not contract the disease. 

The second reactive strategy is moderation of socio-cultural activities that result in mass gathering (festivals, marriage ceremonies, birth naming ceremonies, and funerals) when an outbreak is detected. The third strategy is temporal restriction on movement of carriers. These strategies were described by a male participant and an assemblyman in Vunania as follows:

*The leaders of this community always meet and place bans on the performance of funerals and other communal social-cultural activities when they detect that there is an outbreak of CSM. Through this action, they are always able to control huge gatherings and therefore further spread of the disease. An example is the year 2001 when the leaders temporary placed a ban on the performance of funerals in the community because of a CSM outbreak. Though people could not perform their funerals, this helped a great deal to prevent the spread of the disease.*

*We as community leaders always have a responsibility to protect our people. As a result, we have set up community bye-laws that temporarily place a restriction on the movement of people suspected of having CSM. In this way, we have managed to reduce the spread of CSM for the last ten years, since the last epidemic was experienced.*

#### 3.2.2. Autonomous Adaptation: Modifications in Dwelling Types, Use of Fuel Efficient and Improved Cooking Methods, Sleeping in Open Air

**Figure 2:**
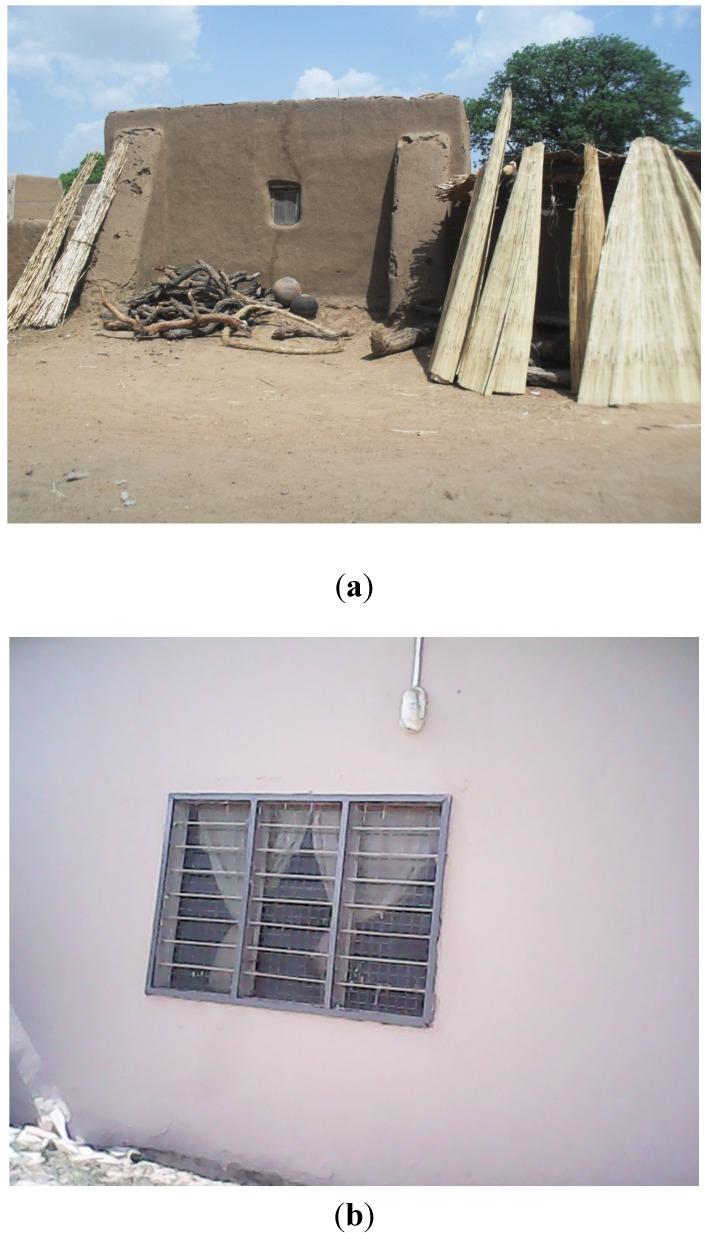
A typical traditional house (**a**) being replaced by a modern house (**b**).

Three main autonomous adaptation strategies were mentioned by FGD participants. These include modifications in dwelling types, use of fuel efficient and improved cooking methods and sleeping in open air. Housing structures in the communities were mainly traditional round houses built of mud, plastered with coal tar or cow dung and roofed with straw or thatch. These traditional houses had limited openings for ventilation, except through the tiny entrances and sometimes a tiny opening which serves as a window. The traditional houses had tiny rooms with high room occupancy rates. These conditions facilitated the spread of CSM epidemics. Most recently, however, people have taken to altering these housing structures to more spacious and ventilated houses to prevent the spread of CSM epidemics (see Figure 2). These improved houses do not only ensure adequate ventilation, through reductions in the amount of heat generated, but also make provision for many rooms that have reduced the room occupancy rates and congestion subsequently. Similarly, the improved housing structures now come with various aeration equipments such as ceiling/standing fans that provide a lot of cool air in the houses. It must however, be pointed out that altering housing structures and improving facilities may not be currently affordable to those who are at highest risk.

*We respect our culture and traditions, including the types of houses we live in. However, the traditional local houses expose us to a lot of heat and diseases like CSM. Based on the recommendation of our community leaders in our community action plans, we decided to modify our houses into cement block houses and zinc or aluminium roofs gradually. Though relatively expensive, we have not regretted because the new houses are more airy and spacious than the previous ones.*

Furthermore, there is now the increased use of improved heating methods in the two communities. Previously, people relied on the traditional stoves that used fuel wood as the main source of energy for cooking and heating. This traditional cooking method generated a lot of heat that worsened the already high temperature, and hence facilitated the spread of CSM. In recent times, communities have resorted to the use of improved clay-based cooking stoves that use a small amount of charcoal. This is because after the clay gets heated up, it can stay hot for as long as two hours. Finally, community members sleep in open and airy places during the dry season to primarily avoid the heat but ultimately the strategy helps prevent the spread of CSM.

#### 3.2.3. Planned Adaptation: Afforestation and Preservation of Sacred Groves in Communities

Afforestation through the growing of trees (mainly teak) both along the boundaries of communities and within houses, and preservation of sacred groves were planned adaptation strategies mentioned by a male participant in Nayagnia and a female participant in Vunania.

*We now grow trees along the boundaries of our communities, and within our houses. We have taken to various afforestation projects to improve upon the natural environment. We have planted teak along the Bolgatanga-Navrongo road just before the Notre Dame Minor Seminary/Secondary School at Nayagnia. We have also planted canopy-top trees that offer various degrees of shades in our backyards. It is these trees that we take cover under during the daytime when temperatures rise so high in the dry season.*

*We have put in place initiatives that help preserve our community sacred groves as an intervention for improving the environment and bringing temperatures down. We revere these sacred groves so we prevent people from going there to cut down the trees.*

## 4. Conclusions

Focus group discussions with residents of two communities (Vunania and Nayagnia), showed a high level of awareness in changes in climatic variables mainly temperature and rainfall. It was generally perceived that duration of the dry season is longer (February to June) than it used to be in the past (March to April). Furthermore, residents are of the view that heat in the dry season is much more severe than what pertained in the past. Regarding rainfall, the view is that the number of rainy days has generally reduced. However, volume of rainfall has seen a lot of variability in recent times. It was stated that prolonged heavy rains are experienced during some seasons, while in others it is only showers and drizzles which last for a very short time. Community members were unanimous in their opinion that flooding is becoming an annual phenomenon as found out by Tschakert, Sagoe, Ofori-Darko, and Codjoe [78], and the situation is aggravated by the opening of the Bagre dam spillway in neighbouring Burkina Faso. 

Although, a few elderly participants ascribed fatal causes (disobedience to gods, ancestors, and evil spirits) to CSM infections, majority of participants rightly linked CSM infections to dry, very hot and dusty conditions experienced during the dry season and more intensely in recent times. They were therefore of the view that future climate change will further exacerbate the CSM prevalence in their communities if appropriate interventions are not put in place.

Based on the findings, it is evident that curbing CSM epidemics in Ghana will require the suite of adaptation options presented in this study. These include—annual mass CSM immunisation; moderation of socio-cultural activities that results in mass gathering and movement restrictions on carriers (reactive adaptation); modifications in dwelling types, use of fuel efficient and improved cooking methods, sleeping in open air (autonomous adaptation); and afforestation and preservation of sacred groves in communities (planned adaptation).
